# Five tips on writing case reports for Japanese generalists

**DOI:** 10.1002/jgf2.395

**Published:** 2020-10-26

**Authors:** Kiyoshi Shikino, Takashi Watari, Masaki Tago, Yosuke Sasaki, Hiromizu Takahashi, Taro Shimizu

**Affiliations:** ^1^ Department of General Medicine Chiba University Hospital Chiba Japan; ^2^ Postgraduate Clinical Training Center Shimane University Hospital Shimane Japan; ^3^ Department of General Medicine Saga University Hospital Saga Japan; ^4^ Department of General Medicine and Emergency Care Toho University School of Medicine Tokyo Japan; ^5^ Department of General Medicine Faculty of Medicine Juntendo University Tokyo Japan; ^6^ Department of Diagnostic and Generalist Medicine Dokkyo Medical University Tochigi Japan

## Abstract

For general physicians, there are two main reasons for writing case reports: to contribute to an academic field and to improve one's own clinical observation, consideration, and diagnostic skills in the longer term. Through our discussions, we have developed five key points, which largely determine whether a case report will be accepted by an academic journal

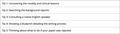

To the Editor:

Writing case reports is important for all physicians, including generalists, to contribute to an academic field^1^ and to improve one's own clinical skills.^2^ However, only a very small proportion of abstracts presented in the official annual conference for general practitioners in Japan are subsequently published as case reports.^3^ Some barriers for Japanese generalists to preparation of case reports were reported as follows: the process of preparing articles, literature search/article acquisition, the time required to prepare articles, disinclination to write articles in English, selection of a journal for publication, and so on.^3^ Through our discussions, we have developed five key points, which largely determine whether a case report will be accepted by an academic journal (Table 1).

**Table 1:** Overview of our 5 tips

Tip 1: Uncovering the novelty and clinical lessons
Tip 2: Searching the background reports
Tip 3: Consulting a native English speaker
Tip 4: Showing a blueprint detailing the writing process
Tip 5: Thinking about what to do if your paper was rejected

Tip 1 *Uncovering the novelty and clinical lessons*. Some clinicians complain that they never get good cases, but this is rarely true. The most essential and first thing to decide in the writing process is to crystallize what are the unique lessons and what are the novel facts of the case,^4^ since the novelty or uniqueness makes the paper appeal to the reviewers.

Tip 2: *Searching the background reports*. Check the background of each question to see whether it can be reported. There is a technique for testing this by assessing a learning point, a key message, and a few problems that are difficult to solve via MeSH term frequencies. If the total number of citations is a few dozen or so, the paper may be publishable somewhere.

Tip 3: *Consulting a native English speaker for editing your case report*. Non–English‐speaking physicians generally find it challenging to start the writing process in English.^3^ They should consult a native English peer reviewer because an adequate use of the English language is one of the most important factors for publication. Although there are some excellent online translation tools, it is not appropriate for a scientific paper format.

Tip 4: *Showing a blueprint detailing the writing process*. Presenting writing process in a flowchart or in a numbered order would make the process easier and actually create a manuscript for the novices. Such clear instructions would also help to spare time for the authors, avoiding the detour of the process.

Tip 5: *Thinking about what to do if your paper was rejected*. Unfortunately, even a carefully written case report can be rejected. But we have to be tough, having a list of alternative journals will facilitate your next journal submission. You can also prepare consent forms in advance, as they may be formatted differently. If there are peer‐review comments, carefully read the comments and use them to rewrite your manuscript.

Finally, generalists should adopt a critical mindset when preparing case reports, incorporating the following questions^5^: What learning points does this case offer to global readers? Will it be helpful for the reader to encounter this case? We hope that these five tips for writing a case report, based on our own academic experience, will help Japanese generalists to submit case reports in the future.

## CONFLICT OF INTEREST

None.
